# Failure points in the early autism identification pathway for children aged 0–5 years: Why screening is not diagnosis

**DOI:** 10.1002/ped4.70079

**Published:** 2026-08-14

**Authors:** Ruslan Kurmashev, Mavile Karaieva

**Affiliations:** ^1^ Department of Biological Sciences Munster Technological University Cork Ireland; ^2^ Independent Researcher Brooklyn New York USA

**Keywords:** Autism screening, Autism spectrum disorder, Clinical pathway, Developmental surveillance, Diagnostic delay, Early identification, Primary care

## Abstract

Early autism identification in children aged 0–5 years is often discussed in terms of screening accuracy, yet consequential delay frequently occurs across the pathway from first concern to referral, diagnostic assessment, and support. This critical narrative review examines early autism identification as a pathway problem rather than as a single testing event. It synthesizes evidence on developmental surveillance, autism‐specific screening, parental and clinician concern, referral conversion, diagnostic waiting, service capacity, inequity, family burden, and pre‐diagnostic support. Screening tools can identify an elevated likelihood of autism and may accelerate diagnosis for some screen‐positive children, but they cannot confirm diagnosis or safely exclude autism when concern persists, and they do not compensate for failures in follow‐up, referral, assessment capacity, or support initiation. Recent evidence supports a cautious interpretation of universal autism screening because diagnostic stability, screening accuracy, and intervention benefit in screen‐detected children remain uncertain. Comparative evidence on the Modified Checklist for Autism in Toddlers, Revised with Follow‐Up suggests context‐dependent performance, including variable sensitivity, low or inconsistent positive predictive value, and age‐dependent accuracy. Multicultural surveillance and implementation studies indicate that adding tools without aligning workflow, language support, follow‐up systems, and service capacity may not improve pathway performance. The review argues that early autism identification should be evaluated through linked quality measures: response to concern, repeated surveillance following negative or ambiguous screening, referral completion, time to diagnostic assessment, support initiation before diagnostic closure, and equity of access. The clinical priority is not a perfect screening instrument in isolation, but faster, more coherent, and more equitable local pathways that translate concern into timely action.

## Graphical abstract

Early autism identification in children aged 0–5 years should be understood as a linked pathway from concern to action. Screening has value, but clinically meaningful identification depends on escalation of concern, referral conversion, timely assessment, and needs‐based support.

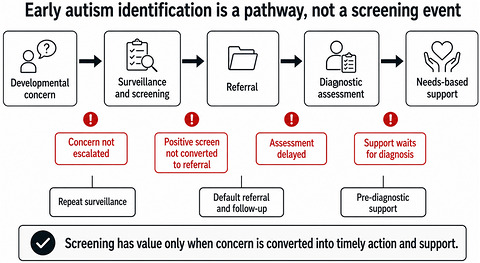

## INTRODUCTION

Early autism identification in children aged 0–5 years is clinically important not simply because it advances the timing of diagnosis, but because it should shorten the interval between first developmental concern and meaningful clinical action. Current pediatric guidance does not treat autism identification as a single testing event; instead, it places autism‐specific screening within a broader process of developmental surveillance, referral, diagnostic evaluation, counseling, and access to early intervention and other supportive services.^1^, ^2^

Despite wider implementation of standardized screening, delays in autism diagnosis remain substantial. A recent meta‐analysis estimated a global mean age at autism spectrum disorder diagnosis of 60.48 months, while a large national health research network study reported an average delay of 26.9 months from first developmental screening to autism diagnosis.^3^, ^4^ These findings suggest that the central weakness in early autism care lies not only in whether risk is detected, but in whether concern or screening findings are translated into timely referral, assessment, and support.

This interpretation is consistent with clinical guidance; the National Institute for Health and Care Excellence (NICE) conceptualizes autism care as a linked pathway of recognition, referral, diagnostic assessment, communication, and follow‐up, rather than as a screening event in isolation.^5^ Framed in this way, early identification may fail at multiple transition points: parental or clinician concern may not be escalated, a positive screen may not lead to referral, referral may not secure timely multidisciplinary assessment, and support may be delayed while families wait for diagnostic closure.

This review therefore examines early autism identification primarily as a pathway problem that includes, but is not limited to, screening performance, focusing on where the 0–5 pathway breaks down after concern has been recognized and on why a screening‐centered model remains insufficient when the downstream system does not respond quickly and coherently.

## APPROACH TO THE CRITICAL NARRATIVE SYNTHESIS

The article was prepared as a critical narrative review examining why early autism identification in children aged 0–5 years fails across the clinical pathway from first concern to referral, diagnostic assessment, and access to support, rather than at the level of screening alone. The review was focused on real‐world clinical pathway performance and was structured around transition points at which momentum may be lost between developmental concern, surveillance or screening, referral, assessment, and the initiation of support. It was critical in the sense that it did not aim only to summarize screening accuracy studies, but to examine the assumptions, implementation gaps, and service‐level failures that limit the clinical value of screening when downstream referral, assessment, and support pathways are delayed or fragmented.

A targeted, iterative literature search was conducted in PubMed/MEDLINE, Europe PMC, and Google Scholar to identify empirical studies, systematic reviews, and other evidence directly relevant to one or more transitions in the 0–5 early autism identification pathway, including developmental concern, surveillance or screening, referral, diagnostic assessment, and initiation of support. Supplementary searches of the NICE and National Health Service (NHS) England websites were used to identify clinical guidance and service‐pathway documents, particularly where explicit organizational detail was needed. The main search emphasis was on literature published from 2010 to April 2026, with a targeted update in June 2026 to identify recent screening and pathway‐implementation evidence relevant to the revision; rather than applying an absolute date cut‐off, earlier papers were retained only when they were foundational to core clinical guidance, early identification, developmental timing, or interpretation of later pathway evidence. Although UK guidance sources were used for service‐organization context, the empirical literature synthesized in the review was drawn from multiple healthcare contexts rather than from a single national system.

The review strategy was structured around six thematic domains that were used to guide both source selection and critical narrative synthesis across the early autism identification pathway. These domains were chosen because they reflect key transition points and barriers at which clinical momentum may be lost, from surveillance or screening through referral, diagnostic assessment, waiting periods, and earlier support‐oriented action. The six domains were: developmental surveillance and autism screening; parent concern and clinician response; referral after concern or positive screening; diagnostic delay, waiting times, and multidisciplinary service capacity; disparities and family burden during the waiting period; and pathway redesign, including earlier action before full diagnostic closure.

Representative search terms included combinations of “autism” or “autism spectrum disorder” with terms related to screening, developmental surveillance, parent concern, clinician response, referral, diagnostic delay, waiting time, multidisciplinary assessment, primary care, early intervention, family navigation, and disparities. Additional studies were identified through backward and forward citation tracking of key guidelines and explicitly defined anchor papers. Anchor papers included major clinical guidance on developmental surveillance and autism identification, large routine‐care or community‐based screening studies, influential studies on referral conversion, diagnostic delay, and pathway transitions, and selected foundational older papers retained for developmental or early‐identification context. Backward citation tracking was used to identify earlier foundational sources, while forward citation tracking was used to identify later replication, implementation, and updated pathway studies.

Priority was given to peer‐reviewed empirical studies, systematic reviews, and major clinical or policy guidance documents directly relevant to preschool‐aged children and to the organization of the identification pathway. When findings conflicted, greater interpretive weight was given to larger routine‐care or population‐based cohorts, longitudinal follow‐up studies, systematic reviews, and studies directly reporting pathway transitions, referral patterns, diagnostic delays, waiting times, or service outcomes. Smaller validation studies, convenience samples, and technology‐focused papers without direct pathway relevance were interpreted more cautiously. No formal risk‐of‐bias tool or meta‐analysis was applied because the review was a critical narrative, and pathway‐oriented rather than systematic. A pooled cross‐system cascade estimate was not attempted because the available studies used different denominators, different pathway definitions, and different service contexts.

The primary age focus of the review was children aged 0–5 years, because this is the period in which developmental surveillance, autism‐specific screening, referral decisions, and early support are most clinically time‐sensitive. However, some guidance and service‐organization studies were broader in age scope. These sources were retained only when they informed pathway structure, referral logic, diagnostic service capacity, or implementation constraints that affect preschool children as part of wider child autism assessment systems. They were not treated as direct preschool‐only evidence unless the original study population or reported subgroup supported that interpretation.

Because the evidence base was drawn from different healthcare systems, the synthesis distinguishes between pathway mechanisms and system‐specific service arrangements. Findings related to missed screening, incomplete referral conversion, diagnostic waiting, and delayed support were interpreted as recurring pathway mechanisms. By contrast, specific programs, time targets, referral structures, funding arrangements, Part C of the Individuals with Disabilities Education Act/Individualized Family Service Plan (IFSP) processes, and NHS service configurations were treated as context‐dependent examples rather than directly transferable models. The purpose of the review was therefore not to propose a single universal diagnostic pathway, but to identify recurrent failure points that require local adaptation within each health system.

## WHAT SCREENING CAN AND CANNOT DO

Autism screening is intended to identify increased likelihood and prompt further clinical action; it is not a stand‐alone act of diagnosis. Current pediatric guidance distinguishes developmental surveillance, formal screening, and diagnostic evaluation as related but separate functions within early childhood care.^1^, ^2^ Screening therefore contributes to early identification only when it is embedded within a pathway that can translate concern or a positive result into referral, assessment, and support.^5^, ^6^

This distinction matters because a positive screening result is not equivalent to an autism diagnosis. NICE states that tools used to identify increased likelihood of autism may support decision‐making, but should not be used either to confirm or to exclude the diagnosis.^5^ Diagnostic assessment still requires developmental history, direct assessment of social communication and behavior, consideration of differential diagnoses, and a broader formulation of functional needs.^1^, ^5^, ^6^

A negative screen should also be interpreted cautiously, because real‐world studies show that autism screening in routine primary care does not perform as cleanly as validation studies may suggest, and false‐negative results do occur.^7^, ^8^ This is consistent with broader reviews emphasizing that no single screening result should override persistent parental concern, clinician observation, or ongoing developmental surveillance.^2^, ^9^

At the same time, uncertainty about the population‐level benefits of universal autism screening should not be confused with evidence that screening has no clinical value. The US Preventive Services Task Force concluded that available tools can identify autism‐related risk in young children, but found insufficient evidence to determine the balance of benefits and harms of universal screening in children for whom no developmental concerns have been raised.^10^ A recent evidence update reached a similarly cautious conclusion: across diagnostic stability, screening accuracy, and intervention outcomes in screen‐detected children under five, the evidence remained inconclusive, with many accuracy studies limited by lack of blinding, incomplete follow‐up of screen‐negative children, and attrition.^11^

More recent comparative evidence on the Modified Checklist for Autism in Toddlers, Revised with Follow‐Up (M‐CHAT‐R/F) strengthens this caution rather than overturning it. A 2026 systematic review of six comparative studies including 12 633 children aged 12–48 months found that M‐CHAT‐R/F performance was context‐dependent, with highly inconsistent sensitivity, variable specificity, persistently low positive predictive value (PPV), and age‐dependent accuracy outside the optimal 16–30‐month window.^12^ Similarly, evidence from one Australian Watch Me Grow cohort showed limited incremental value from adding M‐CHAT‐R/F to tiered developmental surveillance.^13^ These findings support a pathway interpretation: screening tools may identify elevated likelihood, but their clinical value depends on repeated surveillance, correct interpretation, referral conversion, diagnostic capacity, and timely support initiation.

The practical implication, consistent with broader early‐detection and intervention literature, is that screening should be understood as one component of a larger care pathway rather than as the decisive event in early autism identification.^14^ It may help identify children who need closer follow‐up or referral, but it cannot compensate for failures that occur after concern has already been recognized.

## REAL‐WORLD SCREENING EVIDENCE AND EMERGING DIGITAL APPROACHES

A pathway‐centered account does not remove the need to appraise screening tools themselves. Rather, it requires screening performance to be interpreted in relation to implementation fidelity, repeated surveillance, referral conversion, and downstream diagnostic capacity. This distinction is important because commonly used tools do not perform uniformly across routine‐care settings, age windows, and population groups.

In large routine‐care cohorts, the M‐CHAT family of instruments has performed less cleanly than early validation studies suggested. In a primary care network of 25 999 toddlers followed into later childhood, Guthrie and colleagues reported M‐CHAT/F sensitivity of 38.8% and a PPV of 14.6%, indicating that many children later diagnosed with autism had screened negative in toddlerhood.^8^ In another large health‐system cohort of 36 233 toddlers, Carbone and colleagues reported sensitivity of 33.1% and PPV of 17.8%; providers also routinely omitted the M‐CHAT follow‐up interview and showed uneven referral patterns.^7^ Importantly, screen‐positive children in that cohort were diagnosed substantially earlier than children who were not screen‐positive (38.5 vs 48.5 months), indicating that screening may accelerate diagnosis for some children even when overall sensitivity is modest.^7^

More recent comparative evidence supports the same cautious interpretation. A 2026 systematic review of comparative M‐CHAT‐R/F accuracy studies found a complex and context‐dependent performance profile, including highly inconsistent sensitivity, variable specificity, persistently low PPV, and age‐dependent accuracy outside the recommended 16–30‐month window.^12^ This does not mean that M‐CHAT‐R/F lacks clinical value, but it does argue against treating it as a stand‐alone universal solution. Its utility depends on correct age use, follow‐up interview fidelity, repeat surveillance, and the availability of referral and assessment pathways for children who screen positive or remain clinically concerning despite a negative screen.

Evidence for surveillance‐based tools also depends strongly on design, setting, and follow‐up model. In a large Australian community surveillance study, the Social Attention and Communication Surveillance–Revised (SACS‐R) used between 12 and 24 months showed high PPV (82.6%), specificity (99.6%), and negative predictive value (98.7%), but more moderate sensitivity (61.5%). When the preschool follow‐up SACS–Preschool (SACS‐PR) at 42 months was added, estimated sensitivity increased to 96.1%.^15^ This pattern is especially relevant to the present review because it shows that repeated surveillance across early childhood can identify false‐negative cases that would be missed at a single time point. However, recent Australian multicultural cohort evidence also cautions against assuming that adding an autism‐specific screener necessarily improves detection. In the Watch Me Grow cohort, sensitivity and specificity across tool combinations ranged from 51% to 87%, and adding M‐CHAT‐R/F to tiered developmental surveillance did not significantly improve autism likelihood identification compared with tiered developmental surveillance alone.^13^

Digital and app‐based approaches should be interpreted with similar caution. ASDetect is better viewed here as an emerging digital adjunct than as a directly comparable routine‐care screening study. In the evaluation protocol and pilot data, approximately 18% of monitored children were classified as at high likelihood for autism, 71% of parents reported pre‐existing developmental concerns, and pilot PPV was 84%, with a false‐positive rate of 16%.^16^ These findings are promising, but they should not be interpreted as equivalent to validation in unselected routine primary‐care populations.

These data help explain why false‐negative results require explicit clinical attention. A negative autism screen does not safely exclude autism in a child with persistent parental concern, clinician observation of atypical social‐communication development, developmental regression, or later‐emerging difficulties. In the two large routine‐care cohorts discussed above, this corresponds to approximately 61.2% of children later diagnosed with autism not being captured as screen‐positive at the index screening episode in the Guthrie cohort and 66.9% in the Carbone cohort.^7^, ^8^ This does not mean that these children were wholly unrecognized at that time, but it does show that entry into the pathway cannot depend solely on one screening episode and often requires persistent concern, repeated surveillance, recognition of later‐emerging signs, or referral through routes other than an initial positive screen. This is consistent with broader pediatric guidance that developmental surveillance should continue across visits and that a single screening result should not override ongoing clinical concern.^1^, ^2^ In this respect, repeated surveillance is not simply a procedural refinement; it is a practical response to the heterogeneity of autism onset and presentation across early childhood.^15^

The broader implication is that screening performance and pathway performance are related but not interchangeable. Even when a child screens positive, the pathway may still fail if follow‐up interview procedures are omitted, referral does not occur, or families do not reach diagnostic evaluation. Monteiro and colleagues reported that among children who failed one or more autism screens, only 31% were referred for specialist evaluation, despite 18% later receiving an autism diagnosis and 59% receiving another neurodevelopmental diagnosis.^17^ In another large primary care study, only 40.2% of screen‐positive children received at least one recommended referral and only 3.7% received all recommended referrals.^18^ Screening therefore cannot be evaluated only by psychometric performance at the point of detection; its clinical value also depends on whether the surrounding system can convert concern or a positive result into timely assessment and support. Selected real‐world screening, surveillance, and digital‐tool evidence is summarized in Table 1.

**TABLE 1:** Selected reported performance characteristics of commonly cited early autism screening, surveillance, and related digital tools

Tool/model	Study context	Reported performance	Interpretive point
M‐CHAT/F	Universal electronic primary care screening in a pediatric network, 25 999 toddlers followed into later childhood^8^	Sensitivity 38.8%, PPV 14.6%, sensitivity higher with repeated screenings	Routine‐care universal screening missed many children later diagnosed with autism; repeated contacts improved sensitivity.
M‐CHAT	20‐clinic health‐system cohort, 36 233 toddlers^7^	Sensitivity 33.1%, PPV 17.8%, screen‐positive children diagnosed at 38.5 vs 48.5 months, follow‐up interview often omitted	Screen‐positive status was associated with earlier diagnosis for some children, but performance and implementation fidelity remained limited.
M‐CHAT‐R/F comparative evidence synthesis	Systematic review of six comparative studies published 2021–2025, including 12 633 children aged 12–48 months^12^	Highly inconsistent sensitivity, variable specificity, persistently low PPV, and age‐dependent accuracy; performance degraded outside the optimal 16–30‐month window	Recent comparative evidence cautions against treating M‐CHAT‐R/F as a stand‐alone universal screen and supports considering age‐specific, tiered pathways.
SACS‐R/SACS‐PR	Community developmental surveillance, 13 511 children aged 11–42 months^15^	SACS‐R: PPV 82.6%, NPV 98.7%, specificity 99.6%, sensitivity 61.5%, with SACS‐PR sensitivity 96.1%	Repeated surveillance across early childhood can recover false‐negative cases missed at earlier contacts.
PEDS/ASQ‐3/M‐CHAT‐R/F tiered surveillance	Australian multicultural birth cohort, tools used around 18 months, with MSEL and ADOS‐2 assessment at 18–23 months, complete sensitivity/specificity analysis, *n* = 165^13^	Sensitivity and specificity across tool combinations ranged from 51% to 87%; adding M‐CHAT‐R/F did not significantly improve autism likelihood identification compared with tiered developmental surveillance alone.	In one multicultural and socially diverse cohort, adding an autism‐specific screener to developmental surveillance provided limited incremental value and may still miss children needing assessment.
ASDetect	Evaluation protocol and pilot data from concern‐enriched app users, not unselected routine primary care^16^	Approximately 18% at high likelihood, 71% prior parent concern, pilot PPV 84%, false‐positive rate 16%	Emerging digital adjunct evidence from concern‐enriched users, not directly comparable to routine‐care validation studies.

*Note*: Study populations, age windows, follow‐up procedures, ascertainment strategies, and evidence types differed across studies. The table includes both primary cohort studies and one recent comparative evidence synthesis; therefore, it should not be interpreted as a head‐to‐head pooled comparison of screening tools.

Abbreviations: ADOS‐2, Autism Diagnostic Observation Schedule, Second Edition; ASQ‐3, Ages and Stages Questionnaire, Third Edition; M‐CHAT, Modified Checklist for Autism in Toddlers; M‐CHAT/F, Modified Checklist for Autism in Toddlers with Follow‐up; M‐CHAT‐R/F, Modified Checklist for Autism in Toddlers, Revised with Follow‐up; MSEL, Mullen Scales of Early Learning; NPV, negative predictive value; PEDS, Parents’ Evaluation of Developmental Status; PPV, positive predictive value; SACS‐PR, Social Attention and Communication Surveillance‐Preschool; SACS‐R, Social Attention and Communication Surveillance‐Revised.

## WHERE THE PATHWAY FAILS

The screening evidence reviewed above reinforces rather than weakens a pathway‐centered interpretation. Variable sensitivity, low PPV for M‐CHAT‐R/F in several routine or comparative contexts, age‐dependent accuracy, incomplete follow‐up procedures, and the limited incremental value of adding M‐CHAT‐R/F to tiered developmental surveillance in one cohort all indicate that screening cannot function as the decisive clinical event in early autism identification. In practice, early identification begins with parental, professional, or screening‐based concern, but its clinical value depends on whether that concern is converted into referral, diagnostic assessment, and timely support. NICE similarly structures autism identification as a linked process of recognition, referral, and diagnostic assessment rather than as a screening event in isolation.^5^ Framed in this way, early identification may fail even when screening occurs, because major losses often arise at the transitions between stages rather than only at the point of initial detection.

The first breakdown often occurs when parental or clinician concern is noticed but not escalated. Parents commonly report developmental concerns early, yet provider responses may remain reassuring, passive, or observational rather than action‐oriented. Zuckerman and colleagues showed that children later diagnosed with autism spectrum disorder often experienced substantial delay after the first parental conversation with a healthcare provider, and that passive or reassuring responses were associated with longer diagnostic delay.^19^ More recent work also suggests that autism surveillance in primary care remains variable and is influenced by clinician beliefs, child presentation, and practice‐level context, indicating that the pathway can stall before formal screening itself becomes the main problem.^20^

A second breakdown occurs when concern or a positive screening result does not reliably translate into referral. This appears to be one of the most important bottlenecks in the early identification pathway. In a large primary care study, Monteiro and colleagues reported that only a minority of children who screened positive were referred for autism evaluation, despite widespread screening implementation.^17^ Similar concerns have been observed in studies showing incomplete adherence to screening and referral guidance, as well as persistent variation in referral behavior across clinicians and settings.^18^ Evidence from historically underserved populations further shows that a positive primary care screen does not consistently lead to completed diagnostic evaluation, underscoring that screening success and pathway success are not the same thing.^21^ However, implementation studies suggest that this bottleneck is modifiable: electronic decision support, automated reminders, and autism evaluation pathways embedded in primary care can improve screening and referral performance.^22^

A third breakdown appears after referral, when referral does not secure timely multidisciplinary assessment. Here, the problem shifts from frontline detection to service capacity, pathway organization, and workforce structure. A recent national survey of childhood autism assessment services in the United Kingdom found marked variation in diagnostic practice, frequent difficulty meeting recommended timelines, and major service pressures associated with rising referral demand.^23^ These observations are consistent with broader pathway evidence showing that diagnostic waiting times remain long and that the interval between initial developmental screening and autism diagnosis may extend well beyond two years in large health‐system datasets.^4^ Open‐access service studies from the UK further illustrate how variable individual diagnostic journeys can be in practice, including differences in professional time, route complexity, and pathway cost.^24^

The final breakdown is that support is often postponed while families wait for diagnostic closure. Yet developmental need may be evident long before a full autism assessment has been completed. Pediatric guidance emphasizes that developmental concerns should trigger evaluation, counseling, and referral to early intervention or related services as part of ongoing care rather than only after diagnostic confirmation.^1^, ^2^ This point is also supported by service‐access literature, although current intervention evidence is mixed. A pragmatic family‐navigation trial suggests that actively supporting families after a positive screen can increase completion of autism evaluation pathways, but effects on Part C eligibility evaluation and IFSP receipt were limited.^25^ Recent national survey data also suggest that many preschool children with autism still miss very early intervention opportunities, indicating that delay is not only diagnostic but also functional and service‐related.^26^

**FIGURE 1:**
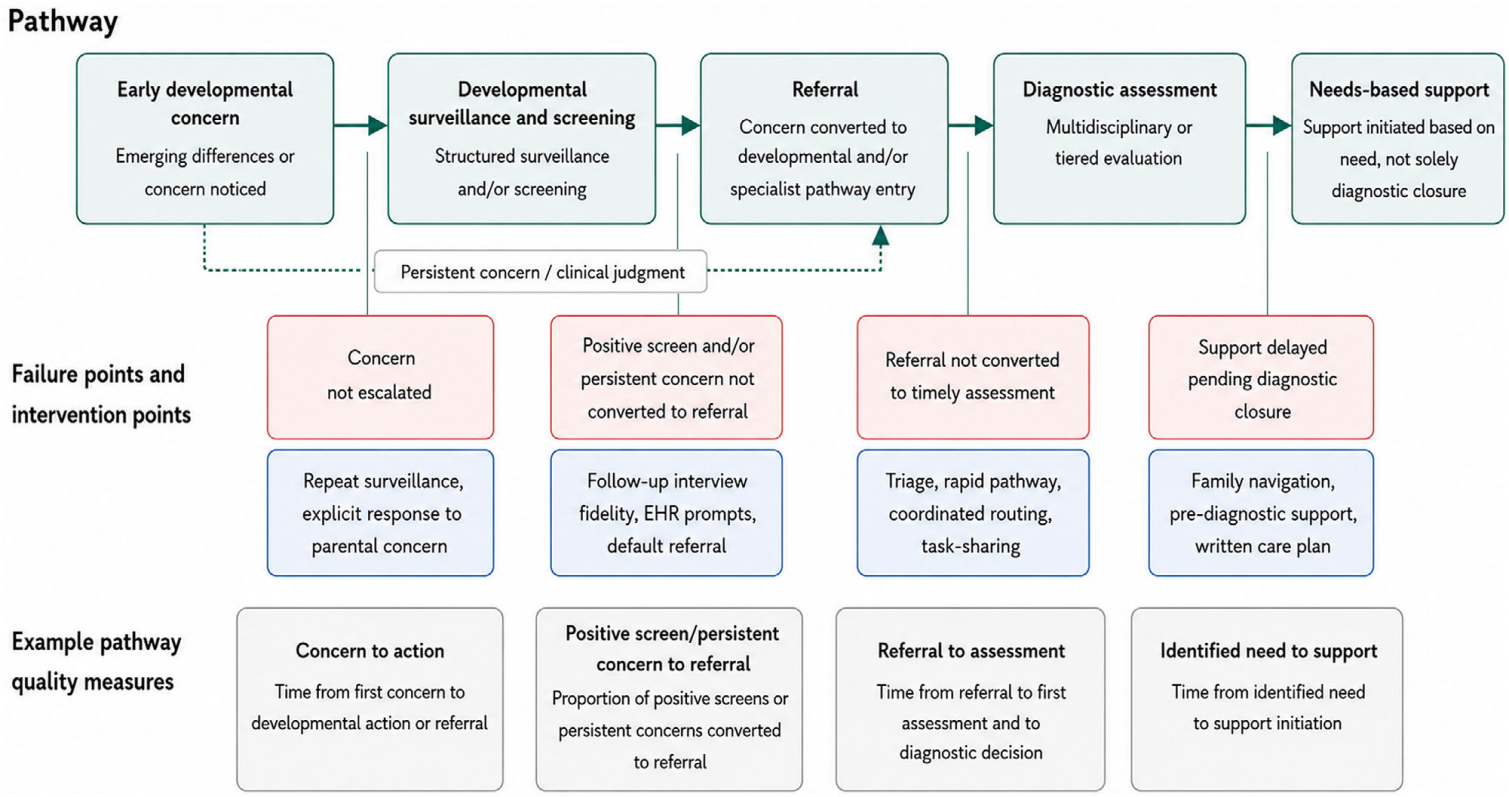
Pathway‐centered model of early autism identification, showing key failure points, intervention points, and example quality measures from developmental concern to needs‐based support.

Taken together, these studies suggest that the central weakness in early autism care lies both in the limitations of early detection and in the ability of the healthcare and service system to respond coherently once concern has been recognized. A child may be noticed but not escalated, screened but not referred, referred but not assessed promptly, or assessed but not rapidly transitioned into support. The pathway fails, in other words, through repeated loss of momentum between stages of care rather than through a single missed event. These recurrent failure points across the early identification pathway are summarized in Figure 1.

## QUANTIFYING PATHWAY BREAKDOWN ACROSS STAGES

Direct pooled estimates of children lost at each transition are rarely defensible because published studies use different denominators, pathway definitions, time anchors, and service contexts. However, routine‐care and service studies do allow stage‐specific quantification of several major handoffs. Table 2 therefore summarizes successive pathway stages, illustrative pathway measures and directly reported data, typical consequences of breakdown, and example corrective actions across different healthcare contexts.

**TABLE 2:** Successive pathway stages, illustrative pathway measures and directly reported data, typical consequences, and possible corrective actions

Pathway stage	Example measure and directly reported data	Typical problem and consequence	Possible corrective action
Initial developmental concern to developmental action or referral	Illustrative quality metric: time from first parental/professional concern to documented developmental action or referral. In a nationally representative US dataset of 1420 children with autism spectrum disorder, passive/reassuring provider responses were associated with longer diagnostic delay than proactive responses,^19^ but directly comparable cross‐system stage‐loss percentages for this early handoff were not consistently reported.	Concern is noticed but not escalated, which delays pathway entry and prolongs diagnostic uncertainty.	Explicit response to parental concern, scheduled follow‐up, repeat surveillance, and a low threshold for referral.
Developmental surveillance and screening	US routine‐care cohorts reported M‐CHAT/F sensitivity of 38.8% and PPV of 14.6%^8^ and M‐CHAT sensitivity of 33.1% and PPV of 17.8%.^7^ In Australian community surveillance, SACS‐R sensitivity was 61.5%, and PPV was 82.6%, rising to 96.1% sensitivity when SACS‐PR follow‐up was added.^15^ Implementation feasibility evidence also shows that screening burden matters: in a Brazilian primary‐care study, 91% of nursing professionals reported that incorporating M‐CHAT into routine child health care overloaded their work, despite reported competence after training.^34^	A single screening encounter may miss children later diagnosed with autism, generate false‐positive burden, or produce screen‐positive results that require careful follow‐up and interpretation. Even when a tool is brief and acceptable in principle, routine implementation may be inconsistent if screening is introduced without protected time, staffing, follow‐up procedures, and downstream assessment capacity.	Follow‐up interview fidelity, repeated surveillance across time points, explicit escalation of persistent concern despite a negative screen, workflow‐integrated screening processes, protected administration time, staff training, and clear follow‐up routes for screen‐positive children.
Positive screen or persistent concern to referral	In one US primary care cohort, only 31% of children who failed a screen were referred for specialist evaluation.^17^ In another network, 40.2% received at least one recommended referral, 3.7% received all recommended referrals, and 11% were referred for comprehensive autism evaluation immediately after a positive screen.^18^	Children with elevated likelihood or persistent concern do not reliably enter specialist or developmental service pathways.	Electronic decision support, default referral prompts, closed‐loop tracking, and explicit monitoring of referral completion rather than referral ordering alone.
Referral to diagnostic assessment	A US national health research network study reported a mean delay of 26.9 months from first developmental screening to autism diagnosis.^4^ In UK service studies, only 17.9% of services reported always meeting NICE timelines,^23^ and one NHS observational study reported a mean time to diagnosis of 375 days and 11.5 h of professional time per child.^24^ Direct referral‐to‐first‐assessment conversion percentages were not consistently defined across studies.	Referral does not convert into timely multidisciplinary assessment, which prolongs uncertainty and delays care planning.	Structured triage, rapid pathways for defined subgroups, capacity planning, coordinated routing, and selective task‐sharing in systems that preserve specialist input for complex cases.
Identified need to support initiation	In intention‐to‐treat analysis, family navigation increased receipt of autism diagnostic evaluation (15/142 [11%] vs. 5/133 [4%], RR 2.81, 95% CI 1.05–7.52) but not Part C eligibility evaluation (36/142 [25%] vs. 31/133 [23%]) or IFSP receipt (32/142 [23%] vs. 29/133 [22%]).^25^ In recent US survey data, only 15% of preschool children with autism were reported to have received early intervention before age 2.^26^	Support is delayed while families wait for diagnostic closure, and inequities in service uptake widen during the waiting period.	Pre‐diagnostic support, family navigation, structured care coordination, and needs‐based action before full diagnostic closure.

*Note*: Table 2 intentionally reports stage‐specific indicators exactly as defined in the source studies. These measures are heterogeneous and should not be interpreted as a pooled cross‐system cascade. Literature gaps remain, particularly for the proportion of children lost between first concern and documented action, and for directly comparable referral‐to‐first‐assessment conversion percentages.

Abbreviations: CI, confidence interval; IFSP, Individualized Family Service Plan; M‐CHAT, Modified Checklist for Autism in Toddlers; M‐CHAT/F, Modified Checklist for Autism in Toddlers with Follow‐up; PPV, positive predictive value; RR, risk ratio; SACS‐PR, Social Attention and Communication Surveillance‐Preschool; SACS‐R, Social Attention and Communication Surveillance‐Revised.

## WHY DELAY MATTERS CLINICALLY

The clinical cost of delayed autism identification is not simply that a diagnostic label is assigned later. It is that the interval between first concern and meaningful action becomes unnecessarily prolonged. When the pathway moves slowly, the consequences include delayed referral, postponed care planning, prolonged family uncertainty, and slower access to developmentally appropriate support.^4^, ^19^, ^20^

This argument should not be overstated as a claim that universal autism screening, by itself, has been proven to improve long‐term developmental outcomes. Recent evidence reviews remain cautious on this point. Grigore and colleagues found that the evidence on screening for autism in young children was not conclusive regarding diagnostic stability, screening accuracy, and the benefits of early interventions in screen‐detected children.^11^ This uncertainty matters because the clinical justification for early identification depends not only on detecting elevated likelihood, but also on whether detection leads to timely, appropriate, and beneficial action.

The clinical cost of delay is therefore best understood at the pathway level. Developmental concerns should trigger evaluation, counseling, and referral to early intervention or related supportive services as part of ongoing care, rather than only after formal diagnostic confirmation.^1^, ^2^ Waiting for complete diagnostic closure before acting risks converting clinical uncertainty into service inaction. The relevant outcome is not simply earlier diagnosis, but earlier movement from concern to developmental formulation, family guidance, referral completion, support planning, and access to needs‐based services.

## TIMING, PERIODICITY, AND DEVELOPMENTAL HETEROGENEITY

The clinical significance of delay is inseparable from developmental timing. Autism‐related social‐communication differences can be detectable within the first year of life for at least some children. Prospective work has shown declining eye fixation from 2 to 6 months in infants later diagnosed with autism, while broader developmental reviews identify early differences in social attention, orienting, gesture, and communication during infancy.^27^, ^28^ Nevertheless, onset is heterogeneous: some children show clearer signs in the second year, some have plateau or regression patterns, and some are not easily distinguishable at a single early time point.^6^, ^28^

This developmental heterogeneity supports repeated surveillance rather than reliance on a single screening encounter. The rationale is not that repeated surveillance or repeated screening eliminates uncertainty, but that repeated contacts create multiple opportunities to revisit parental concern, clinician observation, and earlier negative or ambiguous screening results as the child's developmental profile becomes clearer. Current pediatric guidance recommends developmental surveillance at every health supervision visit, standardized developmental screening at 9, 18, and 30 months, and autism‐specific screening at 18 and 24 months.^1^, ^2^ Evidence from SACS‐R/SACS‐PR also suggests that preschool follow‐up can recover some children missed at earlier contacts.^15^ Recent evidence also supports caution about timing and tool selection: M‐CHAT‐R/F accuracy appears age‐dependent and context‐sensitive, while one multicultural cohort found limited incremental value from adding M‐CHAT‐R/F to tiered developmental surveillance.^12^, ^13^ Repeated surveillance should therefore be understood as a pathway safeguard rather than as a guarantee of detection.

The case for timeliness is strengthened, but also qualified, by intervention evidence. In the Early Start Denver Model trial, toddlers aged 18 to 30 months showed greater gains in IQ, adaptive behavior, and diagnostic severity than children referred to usual community care.^29^ In a later randomized trial, children who began an individualized parent‐implemented intervention at 18 months had greater gains in language, social communication, and daily living skills than those starting the same intervention at 27 months.^30^ However, a recent systematic review and meta‐analysis found low‐to‐moderate certainty evidence that very early interventions do not produce uniformly large or consistent improvements across all clinician‐assessed outcomes by age 3 years.^31^ The implication is not that early action guarantees uniform benefit, but that delay narrows opportunities to begin responsive, developmentally appropriate support while uncertainty is still manageable.

## EQUITY AND HETEROGENEITY OF PATHWAY FAILURE

Pathway failure is not uniform across children, families, or healthcare settings. Delays in autism identification are shaped not only by screening performance or referral practices, but also by broader social, linguistic, cultural, geographic, and service‐level inequities. Diagnostic timing and access may vary according to race and ethnicity, language barriers, socioeconomic constraints, caregiver familiarity with developmental services, geographic location, and local diagnostic capacity.^32^, ^33^

This matters because a pathway‐centered account of early autism identification can become misleading if it assumes that all children move through the same system under the same conditions. Aylward and colleagues argue that disparities in autism diagnosis should be understood within a broader biosocial and systems framework rather than as isolated differences in individual help‐seeking or clinician behavior.^32^ In practical terms, pathway failure may reflect cumulative disadvantage across multiple levels: reduced access to developmental surveillance, lower‐quality communication between families and clinicians, culturally or linguistically mismatched screening processes, delayed referral, longer waits for specialist services, and greater difficulty navigating the period between concern and diagnosis. Equity is therefore not an optional dimension of pathway redesign; it is part of whether the pathway functions at all.

Recent multicultural cohort evidence is consistent with this caution. In the Watch Me Grow Study, tiered developmental surveillance and autism screening were evaluated in a culturally diverse Australian cohort, and sensitivity and specificity across tool combinations were variable. Adding M‐CHAT‐R/F did not significantly improve autism likelihood identification compared with tiered developmental surveillance alone.^13^ This finding should not be interpreted as evidence against autism‐specific screening in multicultural populations. Rather, it cautions that adding another tool may not correct pathway inequity if the tool, administration process, language support, follow‐up system, and service capacity are not aligned with the population being served.

Implementation evidence points to a related service‐level constraint. In a Brazilian public primary‐care feasibility study, most nursing professionals recognized the value of M‐CHAT screening and reported feeling competent to administer it after training, yet routine implementation was still perceived as burdensome.^34^ This does not directly measure inequity, but it illustrates how under‐resourced frontline settings may struggle to make screening actionable without protected time, staffing, training, follow‐up procedures, and referral infrastructure.

These inequities are particularly visible in studies of Hispanic and Latinx families, where barriers to autism diagnosis have been linked to language discordance, limited familiarity with developmental services, structural access constraints, stigma, and poor fit between family needs and service delivery models.^33^ Service‐level studies in culturally diverse underserved communities further suggest that access to early identification can improve when screening and referral pathways are designed around local population needs rather than applied as generic procedures.^35^

Family experience data also suggest that the waiting period is not a neutral interval. For families already facing structural barriers, the period between concern and diagnosis may involve greater difficulty accessing evaluation, understanding next steps, maintaining contact with services, and sustaining engagement during prolonged uncertainty. A pathway that is slow for all families may therefore become disproportionately exclusionary for those already at highest risk of being left behind. Improvement efforts should accordingly be judged not only by average pathway speed, but also by whether they reduce inequities in referral response, specialist access, family navigation, and support during the waiting period.

## A PRACTICAL FRAMEWORK FOR IMPROVEMENT

Improving early autism identification requires more than better screening performance. It requires a pathway that converts developmental concern into action more reliably while also recognizing the capacity constraints under which routine services operate. The critical translational question is therefore not only whether a screening tool, digital adjunct, or service model can perform under research or pilot conditions, but whether the wider pathway can implement it with sufficient fidelity, workforce capacity, and follow‐through in real‐world care.

This distinction is important because discrete improvements at one stage of the pathway may not translate into better outcomes if downstream services remain overloaded or fragmented. In a national UK survey, only 17.9% of services reported always meeting NICE guidance for assessment, referrals rose 115% between 2015 and 2019, and 75.8% reported funding that had stayed constant or decreased.^23^ In an observational NHS pathway study, mean time to diagnosis was 375 days and mean professional time was 11.5 h per child, illustrating why promising models may fail to scale cleanly when workforce shortages, case complexity, and service fragmentation are not addressed.^24^

Implementation evidence from outside the UK points to the same implementation constraint. In a Brazilian public primary care feasibility study, 97 nursing professionals across 21 health facilities administered the M‐CHAT to parents of 267 children aged 16–57 months. Most professionals reported feeling competent to administer the tool after training, but 91% reported that incorporating the M‐CHAT into routine child health care overloaded their routine work, and fewer than half supported continued routine use.^34^ This finding does not speak to diagnostic accuracy; rather, it illustrates that training and tool acceptability are not sufficient for sustainable screening implementation. Screening programs also require protected workflow time, adequate staffing, clear follow‐up procedures, and downstream assessment capacity; without these conditions, implementation may add workload without reliably improving conversion from early concern to assessment and support.

Against this background, four practical priorities are especially important. First, surveillance, screening, and diagnosis should remain conceptually and operationally distinct. Clinical guidance consistently treats these as related but separate functions, and this distinction matters because it helps identify where delay actually occurs.^1^, ^2^, ^5^ A system may achieve high screening uptake yet still perform poorly if concern is not escalated, if a negative screen is treated as definitive despite persistent developmental concern, if positive screening results do not lead to referral, or if referral does not secure timely assessment. For this reason, screening rates alone are weak indicators of pathway quality; more informative measures include whether persistent concern triggers repeat surveillance, referral, and actual entry into developmental or diagnostic services.^1^, ^2^, ^7^, ^8^

Second, referral conversion should be treated as a core pathway performance target rather than as a minor administrative step. One of the most consistent findings in the literature is that concern or a positive screen does not reliably translate into specialist evaluation or early developmental service access. In one primary care study, only 31% of children who screened positive were referred onward, while in a larger network study only 40.2% of children received at least one recommended referral and only 3.7% received all recommended referrals.^17^, ^18^ Services should therefore monitor whether children with persistent developmental concern or screen‐positive findings actually enter evaluation pathways, how long that transition takes, and where loss of follow‐through occurs. Interventions at this step may include electronic decision support, structured referral prompts, and closed‐loop tracking so that referrals are completed rather than merely ordered.^22^, ^25^

Third, support should not be unnecessarily delayed while families wait for full diagnostic closure. Pediatric guidance makes clear that developmental concerns should prompt evaluation, counseling, and referral to early intervention or related services as part of ongoing care, rather than only after a formal autism diagnosis has been completed.^1^, ^2^ In practical terms, this means that functional need should trigger action even when etiological or diagnostic certainty is still evolving. This is particularly important because missed opportunities remain common: in recent US survey data, only 15% of preschool children with autism were reported to have received early intervention before age 2.^26^ In one pragmatic low‐income trial, family navigation increased receipt of autism diagnostic evaluation in intention‐to‐treat analysis (11% vs. 4%) but did not improve Part C eligibility evaluation or IFSP completion, and only 45% of families for whom navigation was indicated actually received it.^25^ Family navigation, structured care coordination, and pre‐diagnostic support may therefore help some families complete evaluations and maintain engagement while diagnostic processes continue, but direct effects on early‐intervention uptake remain limited in current trial evidence and real‐world uptake can be constrained by difficulty contacting families and by linguistic, financial, and organizational barriers.^25^, ^26^

Fourth, pathway redesign should focus on service organization, not only on test performance. National service and pathway studies show why: referrals can rise faster than capacity, multidisciplinary teams may be incomplete, and complex cases require substantial professional time.^23^, ^24^ In the UK, referrals more than doubled in some services while most teams reported funding that had stayed constant or decreased, and the mean diagnostic pathway in one NHS study took 375 days and 11.5 h of professional time per child.^23^, ^24^ Quality‐improvement, rapid‐access, and task‐sharing studies therefore suggest potential value in structured triage, clearer routing, more coordinated cross‐specialty pathways, and, in some settings, selective task‐sharing with trained community pediatricians or primary care clinicians while preserving specialist input for more complex or uncertain cases.^35^, ^36^, ^37^, ^38^, ^39^ Some models have reported shorter waits for defined subgroups, including approximately 6 weeks in an early‐intervention‐linked model and about 51 days in a coordinated neurology‐developmental pathway, although these findings come from specific service contexts rather than directly generalizable system‐wide solutions.^35^, ^38^, ^39^ These findings do not imply that multidisciplinary assessment is unnecessary in all cases. Rather, they suggest that rigid dependence on overloaded specialist models may itself become part of the problem when service demand exceeds capacity.

These priorities can also be operationalized at different levels. At the clinician level, persistent parental or professional concern should trigger repeat surveillance, transparent discussion of uncertainty, and referral or developmental action even after a single negative screen. At the healthcare‐facility level, services need reliable documentation, electronic decision support, closed‐loop referral tracking, and named, culturally responsive navigation or coordination processes for families at risk of pathway loss. At the health‐system level, capacity planning, structured triage, cross‐specialty networks, and protected training for task‐shared assessors are needed to align demand with workforce. At the policy level, financing and accountability should reward pathway timeliness from first concern to referral, referral to assessment, and identified need to support initiation, rather than screening volume alone.^22^, ^23^, ^24^, ^25^, ^35^, ^36^, ^37^, ^38^, ^39^

Taken together, the literature supports a shift from a narrowly screening‐centered to a pathway‐centered model of early autism identification. The key quality question is not only whether a system can detect an elevated likelihood of autism, but whether it can convert concern into timely referral, assessment, and support.

## LIMITATIONS

This review has several limitations. First, it was conducted as a critical narrative review rather than a systematic review or meta‐analysis. That design was suited to a complex clinical and service‐pathway question spanning screening performance, developmental surveillance, referral, assessment, and implementation, but it does not provide the exhaustiveness or reproducibility of a protocol‐driven systematic review.^40^, ^41^ Although the search strategy was made explicit and anchor‐paper guided, study selection and emphasis remained partly interpretive, as is typical of narrative reviews.^41^, ^42^ In addition, no formal risk‐of‐bias instrument or certainty‐of‐evidence framework was applied, so the conclusions reflect structured critical narrative judgement rather than formally weighted evidence grading.^42^

Second, the underlying literature was heterogeneous with respect to populations, age windows, screening instruments, pathway definitions, denominators, follow‐up procedures, and service settings. Although the review focused on children aged 0–5 years, some guidance and service‐organization evidence covered broader child age ranges and was used to interpret pathway structure and capacity constraints rather than to make preschool‐specific prevalence or performance estimates. This was particularly evident across the screening, referral, assessment, and implementation studies drawn from different healthcare systems.^7^, ^8^, ^20^, ^23^, ^24^, ^25^, ^35^ Such heterogeneity limited direct comparison between studies and was one reason pooled cross‐system estimates of children lost at each stage were not generated, since doing so would imply a level of comparability that the available evidence does not support.^43^

Third, the updated evidence on screening and surveillance should be interpreted as supporting a cautious pathway‐centered argument rather than as proving the superiority of any single alternative model. Recent studies strengthen the case against treating M‐CHAT or M‐CHAT‐R/F as a stand‐alone universal solution, but they do not establish a universally optimal replacement pathway. Evidence on age‐specific, tiered, surveillance‐based, or digitally supported approaches remains heterogeneous, and many studies still provide limited information on downstream outcomes such as completed referral, diagnostic timing, intervention initiation, family experience, equity, and long‐term developmental benefit.^11^, ^12^, ^13^ Similarly, implementation evidence indicates that screening can add workflow burden in primary care, but such findings are context‐dependent and should not be generalized without attention to staffing, financing, training, and diagnostic service capacity.^34^

Finally, although evidence was drawn from multiple countries and service models, much of the pathway and implementation literature came from high‐income settings, so the framework proposed here may be more transferable conceptually than operationally. Specific bottlenecks, timelines, and service solutions may not generalize directly across healthcare systems.

## CONCLUSION

Early autism identification in children aged 0–5 years should not be understood only as a question of whether screening tools can detect an elevated likelihood of autism. The evidence reviewed here suggests that clinically consequential failure can arise both at the point of initial detection and across the downstream pathway that should convert concern into action. Screening may identify some children earlier, but its value is limited when false‐negative results are not revisited, positive results are not followed by referral, referral does not lead to timely assessment, or families wait without needs‐based support.

Recent evidence strengthens this pathway‐centered interpretation without supporting an anti‐screening conclusion. Evidence updates and comparative studies continue to show uncertainty and context‐dependent performance in early autism screening, including variable M‐CHAT‐R/F accuracy, limited incremental value of adding M‐CHAT‐R/F to tiered surveillance in one multicultural cohort, and implementation burden when brief screening tools are introduced into routine primary care without sufficient workflow capacity.^11^, ^12^, ^13^, ^34^ These findings do not argue against screening, but they argue against treating screening as a stand‐alone solution.

The clinical priority should therefore shift from the search for a perfect screening instrument in isolation to the iterative design, evaluation, and refinement of faster, more coherent, and more equitable local pathways. Such pathways should preserve the value of developmental surveillance and autism‐specific screening while also addressing follow‐up fidelity, referral conversion, diagnostic capacity, workforce constraints, cultural and linguistic fit, family navigation, and needs‐based support before diagnostic closure. Early autism identification fails, in other words, not at one single point, but across the linked system that is supposed to respond from detection through action.

## CONFLICT OF INTEREST

The authors declare no conflict of interest.
